# Neurofibromin 1 (*NF1*) Splicing Mutation c.61-2A>G: From Aberrant mRNA Processing to Therapeutic Implications In Silico

**DOI:** 10.3390/ijms27031177

**Published:** 2026-01-23

**Authors:** Asta Blazyte, Hojun Lee, Changhan Yoon, Sungwon Jeon, Jaesuk Lee, Delger Bayarsaikhan, Jungeun Kim, Sangsoo Park, Juok Cho, Sun Ah Baek, Gabin Byun, Bonghee Lee, Jong Bhak

**Affiliations:** 1 Korean Genomics Center (KOGIC), Ulsan National Institute of Science and Technology (UNIST), Ulsan 44919, Republic of Korea; ehojune@unist.ac.kr (H.L.); vteddy@unist.ac.kr (C.Y.); s4ngsoo@unist.ac.kr (S.P.); juokcho@gmail.com (J.C.); sabaek@unist.ac.kr (S.A.B.); school04290@gmail.com (G.B.); 2 Department of Biomedical Engineering, Ulsan National Institute of Science and Technology (UNIST), College of Information and Biotechnology, Ulsan 44919, Republic of Korea; 3 School of Medicine, Gachon University, Incheon 21565, Republic of Korea; bonghlee@gmail.com; 4 AgingLab, Ulsan 44919, Republic of Korea; jsw0061@gmail.com; 5 nSAGE, Incheon 21999, Republic of Korea; jaesuklee88@gmail.com (J.L.); delgerbayarsaikhan90@gmail.com (D.B.); 6 Personal Genomics Institute (PGI), Genome Research Foundation (GRF), Osong 28160, Republic of Korea; jungeunkim079@gmail.com

**Keywords:** neurofibromatosis, NF1, alternative splicing, protein isoform, rs1131691100, c.61-2A>G, gene editing, gene expression

## Abstract

The neurofibromin 1 (*NF1*) splice-site mutation c.61-2A>G (rs1131691100) is a rare, pathogenic, autosomal dominant variant that disrupts *NF1* tumor-suppressor function, causing neurofibromatosis type 1 (NF1). Its pathogenic mechanism is poorly understood, and the potential for personalized therapeutic genome editing remains unknown due to the absence of a standard framework for investigating splicing disorders. Here, we performed a comprehensive multi-omics analysis of a de novo c.61-2A>G case from South Korea, integrating short- and long-read whole genome sequencing, whole transcriptome sequencing, and methylation profiling. We confirm that c.61-2A>G abolishes the canonical splice acceptor site, activating a cryptic splice acceptor 16 nucleotides downstream in exon 2. This splicing shift generates a 16-nucleotide deletion, causing a frameshift and premature stop codon that truncates the protein’s N-terminal region. Long-read sequencing further reveals that the mutation creates a novel CpG dinucleotide, which is methylated in the majority of reads. Finally, we assessed therapeutic correction strategies, revealing that CRISPR-Cas9 prime editing is the only viable approach for in vivo correction. This study provides the first comprehensive multi-omics characterization of the *NF1* c.61-2A>G mutation and establishes a minimal framework for precision therapeutic development in silico in monogenic splicing disorders.

## 1. Introduction

Neurofibromatosis type 1 (NF1, OMIM#162200) is a relatively common rare disease (~1 in 3000 births) primarily characterized by multiple benign tumors forming along patients’ peripheral nerves, known as neurofibromas, pigmented café au lait spots on the skin, and Lisch nodules in the eyes [1]. Neurofibromas can range from sparse to plentiful, sometimes covering the entire body, including the face, and vary in size. Among all NF1 symptoms, neurofibromas are arguably the most burdensome. They may cause physical discomfort, as tumors can be painful, itchy, press on nerves, or constrict blood flow [2], as well as aesthetic concerns due to their visibility on the skin. Neurofibromas can also contribute to neurological complications, such as learning difficulties and memory impairments. While many neurofibromas can be surgically removed, they may recur, and surgery on others carries a risk of irreversible nerve damage [2,3].

Neurofibromin 1 (*NF1)* is a large gene with a total of 58 exons, 57 of which are constitutive coding exons, while three are alternatively spliced [4]. NF1 disorder can result from a wide range of lesions in the *NF1* gene, including insertions, microdeletions, exonic nonsynonymous single-nucleotide variants (SNVs), splicing-altering variants, microduplications, or large chromosomal rearrangements such as whole-gene deletions and translocations [5,6,7,8,9]. The disease typically follows an autosomal dominant inheritance pattern, with approximately 50% of cases arising from spontaneous de novo mutations [1]. As a result, about half of NF1-affected offspring are born to genetically healthy parents with no prior family history of the disorder. Such frequent de novo occurrences reflect the *NF1* gene’s mutation rate, which is among the highest reported for any human gene (1:10,000) [5].

NF1 pathogenesis centers on the defective tumor suppressor gene *NF1* and partly resembles cancer [10]. Shared features include a predisposition to tumor formation, diverse causative mutations, and variable symptom severity. In *NF1*, the genomic location and specific region affected by a pathogenic variant may influence clinical outcomes and NF1 protein expression levels [11]. In case of deletions, some studies report a correlation between deletion size and phenotype severity [8,12,13]. Moreover, the low NF1 protein and mRNA expression levels in peripheral blood present a challenge that is also reflected in research [14]. For instance, only one major NF1 mRNA isoform in blood shows a significant correlation between its expression level and phenotypic severity in pediatric patients, although considerable overlap remains between healthy controls and NF1 patients with mild symptoms [15].

All currently available NF1 therapies are limited to symptomatic treatment. Patients do not achieve full recovery and remain at increased long-term risk of malignancies [8,16,17]. Since 2020, two breakthrough drugs have been approved by the Food and Drug Administration for symptom management. Selumetinib (Koselugo), a mitogen-activated protein kinase (MEK) 1 and 2 inhibitor, has shown positive responses in up to 75% of individuals treated in clinical trials [18,19]. Similarly, Mirdametinib (Gomekli), another MEK inhibitor, reduced tumor volume in 42% of patients [19]. Nevertheless, the pathogenic mechanisms of individual *NF1* mutations remain poorly understood at the mRNA level, despite their critical importance for developing personalized treatment strategies.

Here, we analyzed a pathogenic NF1 mutation, rs1131691100, which disrupts *NF1* tumor suppressor function [20]. Despite its rarity among NF1 patients and complete absence in the general population, all three alternative alleles—c.61-2A>G, c.61-2A>C, and c.61-2A>T—have been reported in ClinVar [21] (version 20240331). Due to sample availability, our analyses focus on c.61-2A>G. To date, the effects of the c.61-2A>G mutation have only been characterized using the now-retired single-strand conformation polymorphism and heteroduplex (SSCP/HD) technology [20].

We cataloged NF1 mRNA isoforms in blood in relation to the c.61-2A>G mutation using several widely available bioinformatics tools. We identified their limitations and established a minimal analytical framework for uncovering the pathogenic mechanisms of splicing mutations at the mRNA level. Finally, we elucidated the pathogenic mechanism of the aberrant NF1 mRNA isoform and explored the feasibility of personalized treatment strategies, such as gene editing, as next-generation NF1 therapies.

## 2. Results

### 2.1. Causative NF1 Mutation

**Figure 1:**
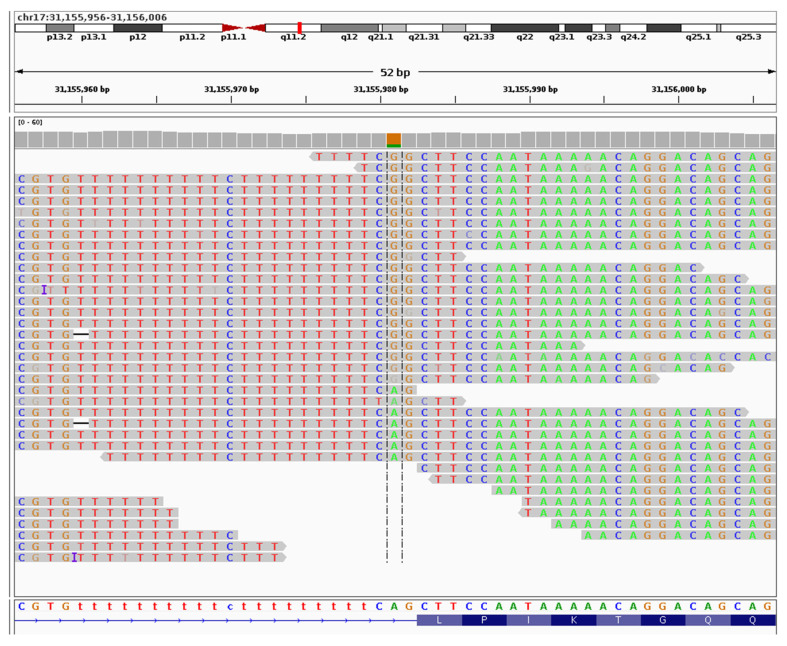
Screenshot of the patient’s DNA reads showing a heterozygous mutation *NF1* c.61-2A>G at the center. The aligned reads were visualized using Integrative Genomics Viewer (IGV) (v.2.17).

We generated three types of omics data from peripheral blood of a male patient from South Korea clinically diagnosed with NF1 (Table S1) and confirmed the diagnosis by identifying the causative c.61-2A>G mutation (Figure 1). Short-read whole genome sequencing (WGS) of the family members (father, mother, and unaffected sibling, sequencing depth of 38×, 45×, and 34×, respectively) did not detect this mutation (Figure S1), confirming its de novo origin (sequencing depth of 41× in the patient). Notably, no other pathogenic SNVs were detected in the patient, and all other variants were deemed clinically irrelevant (Table S2).

In humans this genomic position is highly conserved, with no alternative alleles detected in healthy cohorts (Table S2). The mutation c.61-2A>G is a splice acceptor variant in a canonical exon splice site [22] (https://www.ncbi.nlm.nih.gov/snp/rs1131691100, accessed on 18 August 2025), sandwiched between 20 bp of intronic simple repeats (poly-T) from one side (Table S3) and exon 2 on the other, with only a single base separating it from each of these elements (Figure 1 and Figure S1). The wild type allele ‘A’ at this position (chr17:31,155,981; GRCh38.p14) exhibits relatively high evolutionary conservation, particularly among vertebrates (phyloP7way_vertebrate=1.062; Table S4), implying strong selective pressure. Moreover, c.61-2A>G is consistently annotated as highly pathogenic based on multiple predictive metrics (CADD_phred=24.1, DANN_score=0.992, MutationTaster_score=1; Table S4), suggesting full penetrance. Due to its rarity, even among patient cohorts, this variant is not yet registered in The Human Gene Mutation Database (HGMD) [23] (as of 18 August 2025), although it has been reported in major databases of the National Center for Biotechnology Information (NCBI), such as dbSNP [22] (version 157) and ClinVar [21] (version 20240331).

### 2.2. Additional Structural Variants and Their Pathogenicity

To explore the possibility of pathogenic structural variants (SVs), including large indels and duplications, we pooled SVs obtained from three variant callers—Delly (v1.1.5) [24], Manta (v1.6.0) [25], and pbsv (v. 2.9.0)—(Tables S5–S7) focusing on 24 genes associated with neurofibromatosis or its symptomatic manifestations (*NF1*, *KRAS*, *NRAS*, *SMARCA4*, *HRAS*, *SDC1*, *SDC2*, *PRAS*, *SPRED1*, *BRCA1*, *VCP*, *NF2*, *COQ6*, *LZTR1*, *SMARCB1*, *NOTCH3*, *PDGFRB*, *FAM20A*, *SOS1*, *PDGFRA*, *SDHA*, *SDHB*, *SDHC*, *KIT;* (Table S8). We filtered and ranked these SVs based on predicted clinical impact and manually validated them by visualizing aligned reads in the SV regions using IGV (v.2.17) [26].

We detected no exonic or reportedly pathogenic SVs (Tables S5–S7). Following manual curation, we identified several likely benign intronic *NF1* SVs, each shared between the patient and one of the parents. These included a 21-base pair (bp) heterozygous deletion in the patient’s intron 36, called by Manta (v1.6.0) [25] (Table S6, Figure S2), and a 350 bp LINE1 repeat deletion in the intron 38, called by Manta (v1.6.0) [25] and pbsv (v. 2.9.0) (Tables S6–S7 and S9, Figure S3).

Apart from *NF1* variants, only one relevant, likely benign SV was confirmed: a homozygous ~77 bp deletion in the patient’s *KIT* gene in intron 7 (Figure S4, Tables S6 and S7). This deletion was detected by all three SV callers in both short- and long-read data, with minor differences in start and end positions) (Figure S4, Tables S5–S7). Notably, it was also present in all unaffected family members in a heterozygous state (Figure S4, Tables S5–S7). The deleted DNA sequence corresponds to ALU repeats (Table S9) but lacks population allele frequency and pathogenicity annotations (Tables S5 and S6).

### 2.3. Estimating NF1 mRNA Expression and Alternative Splicing

**Figure 2:**
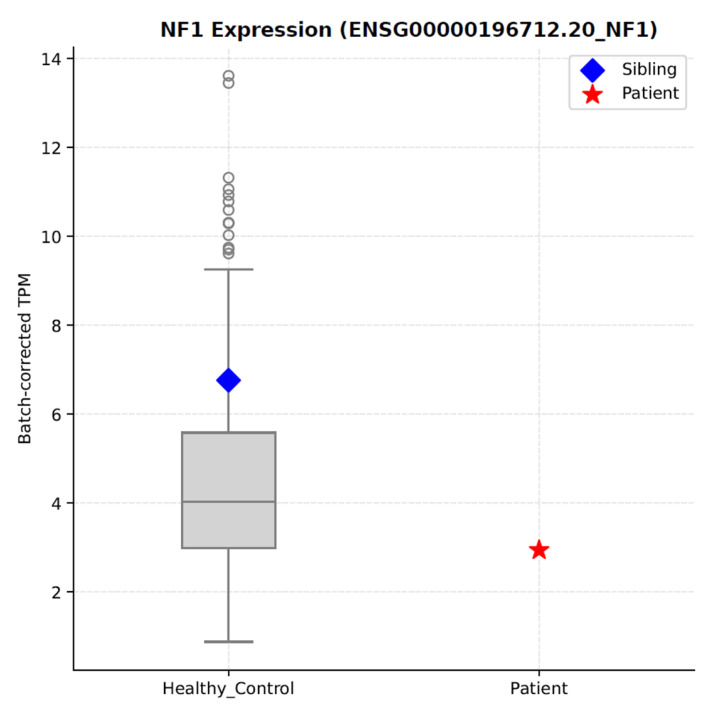
*NF1* gene expression levels in peripheral blood. Normalized RNA expression levels in Transcripts Per Million (TPM) in the Korean general population (healthy controls) compared with an NF1 patient carrying the c.61-2A>G variant. The patient carrying the c.61-2A>G variant shows reduced *NF1* expression (TPM = 2.94) compared to healthy controls (mean ± SD: 4.43 ± 1.97 TPM; Z = −0.76, *p* = 4.50 × 10^−1^), corresponding to the 23.8th percentile of the control distribution.

Our quantified normal expression range indicates that *NF1* expression in whole blood varies widely among the general adult population and is little dependent on age (*p* = 0.0068) but not on sex (*p* = 0.443), based on RNA-seq data from the Korean Welfare Genomics Project (WGP) [27] and Korea10K [28] (Figure 2, Figure S5). The patient carrying the c.61-2A>G mutation exhibited *NF1* expression comparable to the lowest values observed among controls, indicating that this variant does not substantially reduce overall *NF1* expression in blood (Figure 2).

However, this analysis did not identify any high-confidence patient-specific *NF1* transcripts (Table S10). Therefore, we assessed alternative splicing events and *NF1* mRNA isoform diversity using four RNA-based tools from the DICAST [29] pipeline (Table S11) and two DNA-based prediction tools, SpliceAI (v. 1.3.1) [30] and Pangolin (v. 1.0.1) [31]. The DNA-based tools predicted that c.61-2A>G would abolish use of the canonical splice acceptor site (at genomic position chr17:31,155,983), with similar confidence (SpliceAI = 0.98; Pangolin = 0.86) that the next available ‘AG’ splice acceptor lies ~18 bases downstream (Table S12).

Among tested DICAST [29] tools, only Whippet (v. 0.11.1) [32] detected six unique *NF1* isoforms in the patient’s sample (Table S11) and revealed an overall enrichment of alternative splicing events in the patient (n = 39) compared with the healthy sibling (n = 30) and other controls. However, five of the six unique *NF1* isoforms—three instances of multiple exon skipping (MES) and two instances of alternative 3′ splice site (A3) usage—did not involve introns or exons adjacent to the c.61-2A>G mutation (Table S11). Only the remaining predicted A3 event corresponded to the expected genomic location and the splicing consequence previously reported [20].

In parallel, DNA-based splicing prediction tools SpliceAI (v. 1.3.1) [30] and Pangolin (v. 1.0.1) [31] both identified c.61-2A>G as the *NF1* variant with the strongest impact on splicing, scoring it 4–5-fold higher than any other *NF1* variant in the patient (Table S12). SpliceAI (v. 1.3.1) [30] predicted five additional variants with only mild effects on *NF1* splicing (scores 0.05–0.18), while Pangolin (v. 1.0.1) [31] identified four such variants (Table S12). These additional variants, however, were deemed clinically irrelevant due to their intronic location, presence in the healthy general population, and absence of pathogenicity annotations in ClinVar (version 20240331) database [21].

### 2.4. Methylation and the Pathogenic Mechanism of NF1 c.61-2A>G

We observed that the c.61-2A>G mutation created a novel CpG site, which was methylated in ten of the twelve long-read DNA reads (Figure S6). According to the UCSC genome browser [33] (version: GENCODE V49, Ensembl v115), the genomic location of the newly methylated cytosine (chr17:31,155,980) overlaps with two predicted Irf1 transcription factor binding sites on the opposite strand (Table S13). However, we found no evidence that this position corresponds to an open-chromatin region (Table S13), consistent with our previous observation of no exon 2 skipping or intron 1 retention events, suggesting that the observed methylation adds no additional complexity to the pathogenic splicing mechanism of c.61-2A>G.

**Figure 3:**
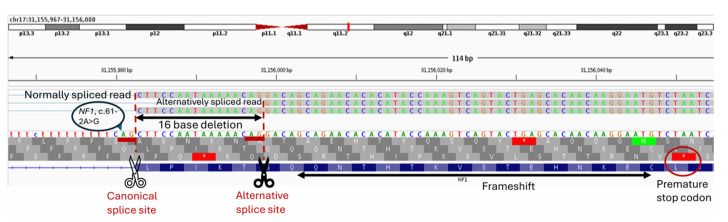
Alternative splicing mechanism of *NF1* c.61-2A>G illustrated using DNA reads. The mutation c.61-2A>G is denoted by blue bubble. Premature termination codons in all three reading frames are denoted as red blocks with a white star. The premature termination codon in the relevant reading frame is additionally denoted by a red bubble. The aligned reads were visualized using Integrative Genomics Viewer (IGV) (v.2.17).

Notably, the disruption of the canonical splice acceptor site by the c.61-2A>G mutation, and the resulting use of an alternative splice site downstream, not only creates the 16-base deletion reported in the previous study [20], but also elicits notable secondary effects (Figure 3). These include a frameshift spanning 17 amino acids before the premature termination within exon 2, truncating it by an additional 26 amino acids (Figure 3, Table S14).

**Figure 4:**
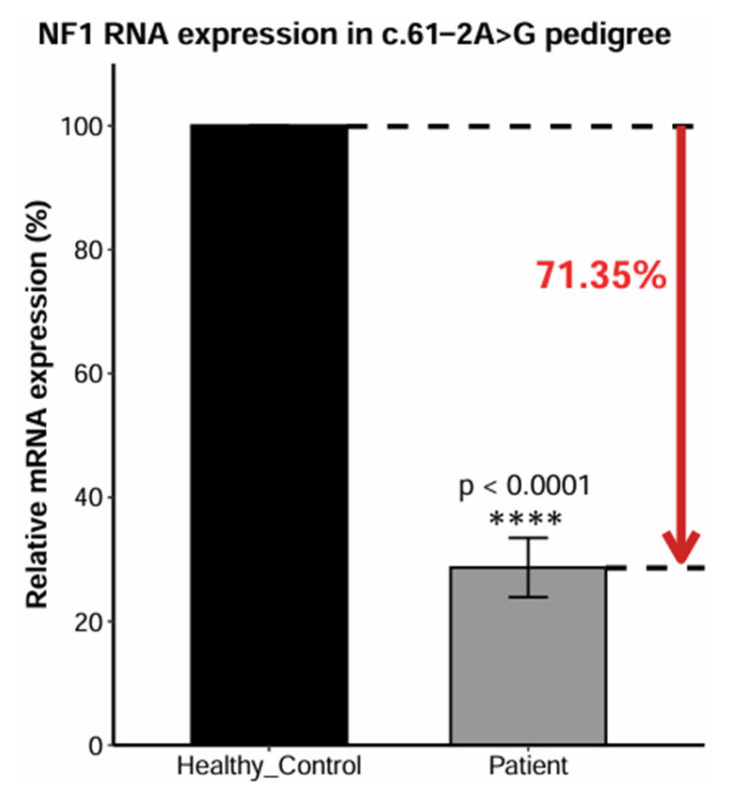
The *NF1* mRNA quantification in the NF1 c.61-2A>G pedigree using RT-qPCR. The results show that *NF1* c.61-2A>G mutation significantly reduces relative mRNA expression suggesting NMD (Table S15).

Although, the deletion and its secondary consequences are confined to exon 2, which corresponds to N-terminal region lacking known *NF1* functional domains [34], the presence of a premature termination codon is expected to predispose the transcript to nonsense-mediated mRNA decay (NMD). To investigate the cause of dysfunction of this *NF1* isoform, we quantified *NF1* mRNA expression across the patient’s family pedigree using quantitative real-time PCR (RT-qPCR) (Figure 4 and Figure S7, Table S15). The observed 71.35% reduction in the patient’s relative NF1 mRNA levels (Figure 4, Table S15) is consistent with haploinsufficiency caused by NMD of the severely truncated transcript [35].

### 2.5. Prospects for Therapeutic NF1 c.61-2A>G Base Editing and Prime Editing

Given that *NF1* c.61-2A>G is an autosomal dominant pathogenic SNP underlying a monogenic disorder, we evaluated the feasibility of its therapeutic (G>A) correction via base- and prime-editing, based on sequence analysis and available literature. The patient’s DNA sequence within ±20 bases of the target mutation contains multiple motifs recognized by different nucleases as either protospacer-adjacent motifs (PAM) or transposon-adjacent motifs (TAM) (Figure S8, Table S16).

Considering factors such as the editing window and reported base-editor activity in mammalian cells, we identified a single Cas12a PAM site (5′-BAAA-3′) located nine bases downstream of the target mutation at the 3′ end as the most suitable candidate for base editing (Figure S8). However, the target mutant guanine (G) lies immediately adjacent to a wild-type G within the same splice-acceptor site, posing a critical limitation for Cas12a base editing (Figure 1, Figure S8). Due to unspecific nuclease activity across the editing window, the intended G>A correction would be accompanied by a bystander G>A substitution at this neighboring base, creating another pathogenic variant (rs1263745475; chr17:31,155,982 G>A), reported in ClinVar [21] (version 20240331) as causative for NF1.

This limitation can be avoided by using CRISPR-based prime editing, an emerging clinical genome editing approach that relies on a distinct mechanism from base editors [36,37]. PE-designer [38] (http://www.rgenome.net/pe-designer/, accessed on 22 August 2025) predicts that an nCas9 (5′-NGG-3′) prime editor could correct the *NF1* c.61-2A>G (Table S17) without introducing the previously mentioned bystander edit.

## 3. Discussion

**Figure 5:**
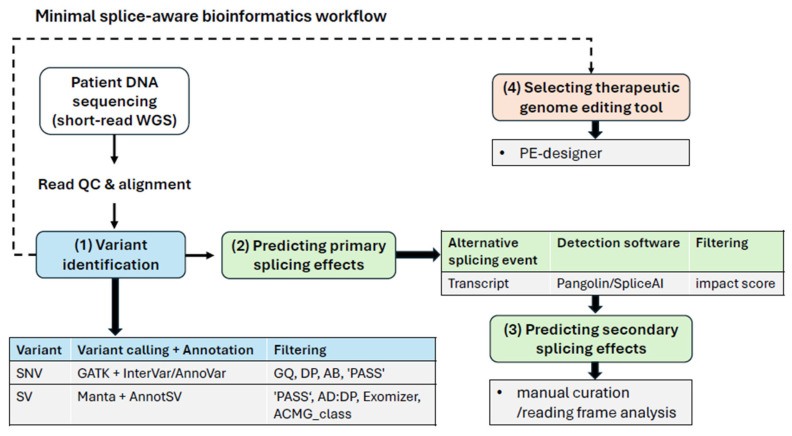
Minimal bioinformatics workflow for analyzing monogenic splicing disorders.

Overall, short-read DNA sequencing was sufficient to capture both the causative NF1 variant and the resulting alternative splicing event, outperforming several RNA-based splicing prediction tools. However, neither RNA- nor DNA-based bioinformatic tools predicted the secondary consequences of the 16 bp deletion—namely, the frameshift affecting 17 amino acids and the additional 26-amino-acid truncation—thereby missing important functional consequences. These effects required an additional reading frame analysis, highlighting a significant limitation of current splice prediction software that must be addressed through either detailed manual curation or additional advanced bioinformatic screening (Figure 5). Consequently, this case study contributes to the broader understanding of splicing–altering mutations by identifying limitations in current RNA- and DNA-based approaches and proposing a minimal analysis workflow: (1) identify pathogenic variants, (2) predict their primary splicing effects, (3) predict their secondary consequences, and (4) select an appropriate genome editing tool—all based solely on short-read DNA sequencing (Figure 5).

By design, many short-read-based gene expression analyses rely on annotated reference transcripts, quantifying known transcripts, which may lead to under-detection of rare or novel isoforms [39,40], such as those that may arise in rare disease patients. Furthermore, low RNA stability during sample preparation and extraction [41], as well as technical limitations in short-read alignment [42], may introduce artifacts, underscoring the importance of manual curation of detected mRNA isoforms. Conversely, limited isoform detection sensitivity can lead to missed splicing events, as demonstrated by three of the four short-read RNA-based splicing prediction tools we tested: IRFinder (v. 1.3.1) [43], EventPointer [44], and SplAdder (v. 2.4.3) [45] (Table S11). Only Whippet (v. 0.11.1) [32] successfully detected the relevant isoform, but with poor specificity, yielding a 5:1 false-positive ratio that required extensive manual curation. Interestingly, DNA-based splicing prediction tools, SpliceAI (v. 1.3.1) [30] and Pangolin (v. 1.0.1) [31], proved advantageous over RNA-based approaches. Both correctly identified the alternative splicing event, provided sufficient context, and ranked the causative NF1 variant as the most impactful on splicing (Table S12). Moreover, this approach enables simultaneous testing of multiple variants and straightforward isoform filtering based on splicing impact, quantified on a numerical scale from 0 to 100 (Figure 5).

While short-read WGS is the gold standard for SNV identification, it is typically suboptimal detecting SVs due to sequencing depth fluctuations and potential short-read alignment artifacts [46]. To circumvent these inherent limitations, we employed HiFi long-read sequencing to confirm SVs in genes associated with neurofibromatosis (Table S8). Nevertheless, our results—particularly Manta (v1.6.0) [25] calls (Table S6)—indicate that short-read DNA sequencing at moderate depth (average 40× in our study, see Table S1) can still provide a baseline SV analysis suitable for diagnostic workflows (Figure 5). The main limitation we encountered in SV analyses was not detection or replicability, but accurate estimation of SV pathogenicity. For instance, the two clinical impact scoring systems we used, Exomiser [47] and ACMG_class, often disagreed in scoring intronic SVs (Tables S5–S7), a discrepancy exacerbated by the lack of population frequency and ethnicity-specific annotations. Although we did not identify any reportedly pathogenic SVs in this NF1 case study (Tables S5–S7), we still recommend including SV assessment in diagnostic workflows, as large indels— particularly deletions that may span entire genes [8,48]—have been reported as causative variants in rare diseases [9]. Although we identified one homozygous deep-intronic ALU SV in the patient’s *KIT* gene (Figure S4), and given that exonic *KIT* deletions are common in neoplasms [49,50,51], we found no reports of ALU-mediated breakpoints confined to *KIT* exons or introns. By contrast, pathogenic ALU-mediated deletions have been well documented in other genes [52,53,54,55].

While patients with *NF1* c.61-2A>G variant may not benefit from direct DNA base editing due to the risk of pathogenic bystander edits, in vivo prime editing represents a theoretically promising alternative that should be further validated beyond in silico settings. Other therapeutic strategies, such as mRNA-based overexpression of the wild-type NF1 allele, could be broadly applicable across NF1 patients by compensating for the defective copy. Nevertheless, whenever feasible, personalized therapeutic approaches are likely in the best interest of patients with NF1 and other rare diseases. Notably, DNA editing provides a permanent correction [56], whereas approaches that modulate mRNA expression are inherently transient and would typically require continuous dosing [57], potentially resulting in lifelong dependency on treatment. Since these treatment strategies are based on entirely different mechanisms, the choice has implications for delivery method and long-term patient management: mRNA is commonly delivered encased in lipid nanoparticles [58], and its modulation is prone to immunogenicity [59] as well as stability issues (intracellular stability and translational efficiency) [59], whereas DNA editing carries risks of off-target effects [60] and severe immune reactions to its viral vector (adeno-associated virus) at high concentrations [61,62,63]. Both modalities face practical challenges in neurological disorders, such as NF1, including crossing the blood–brain barrier [64,65,66] and accessing the peripheral nerve sheath [65,66]. However, important caveats exist: DNA-editing strategies (such as CRISPR/prime editors and base editors) require large vectors that can deliver editing systems into the cell nucleus, in order to correct the genomic DNA [67], whereas mRNA-based therapies act in the cytoplasm and thus avoid the nuclear-import barrier [68]. On the downside, the frequency and continuity of mRNA modulation therapies may in part be dictated by protein turnover rates [67], which for NF1 are not well established [69]. Few sources, using largely irrelevant cell lines (renal carcinoma cell line RCC4), estimate NF1 protein half-life at ~35–48 h, with a drastic decrease under cellular stress conditions [69].

There are three alternative alleles reported in dbSNP [22] (version 157) and ClinVar [21] (version 20240331) under the same ID, rs1131691100, which are suggested to share the same pathogenic mechanism, although the diseases they predispose to differ according to ClinVar [21] database (version 20240331). In principle, different substitution types at this genomic position may elicit distinct epigenetic effects. For instance, c.61-2A>C may be more prone to exon skipping or intron retention than c.61-2A>G because the newly created CpG site resides within the splice-acceptor site, making it susceptible to direct methylation. In contrast, our findings indicate that c.61-2A>G generates a novel CpG site one base upstream of the splice-acceptor site but does not induce intron retention or exon skipping, whereas c.61-2A>T would not create any novel CpG site.

Genome-wide analyses have shown that CpG methylation near splice junctions often correlates with exon inclusion levels [70]. While this provides a mechanistic rationale, we found no peer-reviewed evidence directly linking CpG methylation to *NF1* splice-site regulation, which adds novelty to our observation. Mechanistically, DNA methylation can modulate exon definition and alternative splicing through recruitment of methyl-CpG binding proteins and local chromatin remodeling [70]. However, functional validation is required to establish causality between splice-site mutations, their methylation state, and downstream transcriptomic effects in disorders caused by alternative splicing, including relevant cases of NF1, a topic that is unfortunately beyond the scope of our study.

In this study, while attempting to investigate the pathogenic mechanism of the *NF1* c.61-2A>G mutation beyond in silico bioinformatic analyses, we encountered several critical experimental limitations, including limitations in the analysis of NF1 by Western blot. The mutant NF1 protein, severely truncated due to the premature termination codon, is suboptimal for Western blot analysis because of its small size (one and a half exons; ~4 kDa) and the absence of commercially available antibodies capable of recognizing the mutant NF1 epitope. To circumvent limitations related to mutant NF1 protein size, we quantified downstream protein expression in the MAPK pathway (ERK, p-ERK, and β-actin), where NF1 acts as a suppressor, using Western blot. However, this analysis was not informative, a result that we attribute to the old age of the blood sample. We also designed wild-type *NF1* primers to quantify *NF1* expression at the mRNA level, which confirmed a decrease in *NF1* mRNA expression in the patient, explaining haploinsufficiency and suggesting involvement of NMD in the pathogenic mechanism of *NF1* c.61-2A>G (Figure 4, Figure S7, Table S15).

Low *NF1* gene expression in peripheral blood posed a major challenge in this study. Although blood is a common source for health-related biomarkers [71], it provided insufficient resolution for *NF1* gene expression and most RNA-seq-based isoform analyses (Figure 2). Neurofibromas, Schwann cells, or skin fibroblasts would provide more clinically relevant *NF1* expression profiles, yet these samples were not available. If the patient exhibited segmental NF1, it remains uncertain whether the causative mutation could be detected in the peripheral blood leucocyte DNA at all. These observations raise an important question about which tissue types are most appropriate for NF1 diagnosis and monitoring, particularly if multi-omics approaches become fully integrated into routine healthcare.

## 4. Materials and Methods

### 4.1. Informed Consent and Sample Collection

This study is a part of Korea10K project, which is an ongoing extension of WGP [27], Korea1K [72] and Korea4K [73] projects that focused on clinically healthy members of general population. Therefore, the informed consent acquisition and sample collection, as well as DNA and RNA sequencing, were carried out via Korea10K project following short-read DNA and RNA protocols established [27,72,73].

### 4.2. Neurofibromatosis-Relevant Gene Identification

Beta-version of web Phenomizer-Orphanet [74] (currently deprecated) was used to identify genes relevant in Neurofibromatosis. NF1 protein–protein interaction network was obtained from String database [75] (v.11.0b) (https://string-db.org/) (Table S6).

### 4.3. Short-Read DNA WGS

In short, for short-read sequencing, DNA was extracted from peripheral blood using DNeasy Blood & Tissue kit (Qiagen, Hilden, Germany), and sequencing libraries were prepared using the TruSeq Nano DNA sample prep kit following manufacturer protocol. Sequencing was performed using Illumina Novaseq platform. Read quality check was performed using fastp (v. 0.23.1) (https://github.com/OpenGene/fastp (accessed on 19 January 2026).), default fastp (0.23.1) filters, and thresholds were used to remove low quality reads (Table S1). Read alignment was carried out using BWA-MEM [76] (ver. 0.7.16a) followed by variant calling using Genome Analysis Tool Kit (GATK) [77] (ver. 4.6.2) resulting in approximately 3.9 million SNVs per sample (Table S1).

### 4.4. Short-Read RNA Whole Transcriptome Sequencing

For short-read RNA sequencing, total RNA was extracted using PAXgene blood RNA kit from Qiagen (Hilden, Germany) according to manufacturer’s instructions. The extracted RNA quality was assessed using Bioanalyzer system (Agilent, Santa Clara, CA, USA). The mRNA was purified from total RNA using polyA selection followed by fragmentation. RNA-sequencing libraries were constructed from double-stranded cDNA using an Illumina TruSeq Stranded mRNA Library Prep Kit and sequenced on the Illumina Hiseq2500 platform. Read quality was evaluated using fastp (v. 0.23.1) (https://github.com/OpenGene/fastp), default fastp (0.23.1) filters, and thresholds were used to remove low quality reads (Table S1). Mapping was carried out using STAR [78] (v. 2.7.3) (https://github.com/alexdobin/STAR (accessed on 19 January 2026)), resulting in an average depth of coverage of 3.4× and 3.08× in NF1 patient and healthy sibling samples, respectively (Table S1).

### 4.5. Long-Read DNA WGS

HiFi library was prepared according to SMRTbell prep kit 3.0. After fragmentation of genomic DNA, fragments underwent damage repair, end-repair, and A-tailing. The SMRTbell library was produced by ligating universal hairpin adapters onto double-stranded DNA fragments, followed by exonuclease treatment and AMPure PB beads purification. After the exonuclease and AMPure PB beads purification steps, sequencing primer was annealed to the SMRTbell templates, followed by binding of the sequence polymerase to the annealed templates. The library was checked with Qubit^®^ 2.0 Fluorometer (Thermo Fisher Scientific) for nucleic acid quantification and with Bioanalyzer (Agilent) for fragment size distribution.

### 4.6. Short-Read DNA Analysis

Post-variant calling QC filtering was conducted using the following thresholds: GQ:30, DP:20, AB:0.25, filter: ‘PASS’. Variant annotations were carried out using InterVar (v. 2.2.1) [79] and filtered for ‘Pathogenic’ variants retaining final 0~3 pathogenic mutation candidates per sample (Table S1). Repeats preceding rs1131691100 were identified using RepeatMasker Web Server open-4.0.9 (Dfam: 3.0). Structural variants were called using Delly (v1.1.5) [24], Manta (v1.6.0) [25] filtered based on read support (minimum 10), annotated using AnnotSV [80] web server with neurofibromatosis-related HPO term identifiers: HP:0007524, HP:0005220, HP:0001067, and then ranked based on their Exomizer [47] score > 0.7 and ACMG_class score ≥ 3. Alternative splicing scenarios were additionally investigated using SpliceAI (v. 1.3.1) [30] and Pangolin (v. 1.0.1) [31] with default settings.

### 4.7. Short-Read RNA Analysis

Short-read RNA expression analysis of WGP [27] and Korea10K [28] whole-RNA data (Table S18) was conducted using R package RSEM (v1.3.3) [81] for transcript quantification, generating TPM (Transcripts Per Million) values for each gene. The batch effect removal was conducted using ComBat [82] on log2 transformed TPM values. Prior to batch correction, genes with low expression (TPM ≤ 1 in more than 90% of samples) were filtered out, resulting in 16,526 genes for downstream analysis. For *NF1* expression analysis, the patient and unaffected sibling were compared against 609 healthy controls. Statistical significance was assessed by calculating Z-scores, representing the number of standard deviations from the control mean. Two-tailed *p*-values were derived from the standard normal distribution. The 95% confidence interval for the control population was calculated using the t-distribution. To assess potential confounding effects of demographic factors, one-way ANOVA was used to test for age-related differences across six age groups: (1) 20–30 (F:106, M:96); (2) 31–41 (F:58, M:52); (3) 42–52 (F:47, M:47); (4) 53–60 (F:40, M:29); (5) 61–69 (F:33, M:29); and (6) 70–84 (F:36, M:36) years, and an independent two-sample *t*-test was used to compare expression between males and females. All statistical analyses were performed using Python (v. 3.12) package SciPy (v.1.11.) [83] with *p*-values < 0.05 considered statistically significant.

For RNA isoform interpretation, we used Ensembl database (release 115) [84] to identify transcripts and their corresponding annotations, then confirmed which exons the transcripts cover using BLAST/BLAT Alignment utility in Ensembl (release 115) [84] web database (based on the genomic coordinates shown with output alignments) and IGV [26] (v.2.17). For computational isoform diversity prediction from short RNA reads, we utilized DICAST [29] pipeline that itself contains tools Whippet (v. 0.11.1) [32], IRFinder (v. 1.3.1) [43], EventPointer [44] and SplAdder (v. 2.4.3) [45]. For DICAST [29] isoform analysis, depending on the tool, we used 12–167 random WGP [27] samples as controls and additionally a patient’s healthy sibling’s sample (Table S10) to identify isoforms unique to *NF1* case and *NF1* c.61-2A>G mutation specifically.

### 4.8. Methylation Analysis

Differential Methylation Region (DMR) analysis was conducted on long-read DNA data. The 5mC sites were predicted using Jasmine (v.1.1.1) (https://github.com/PacificBiosciences/jasmine/ (accessed on 19 January 2026).) and converted to BigWig format with pb-CpG-tools (v.2.3.1), which was used for IGV [26] (v.2.17) visualization.

### 4.9. Long-Read DNA Analysis

We extracted 4709,662 HiFi reads with read quality > Q20 using pbccs (v.6.4.0) and aligned them to human genome reference GRCh38 using pbmm2 (v.1.13.1) achieving an average depth of coverage of 29.3× (Table S1). SV calling was carried out employing pbsv (v.110) and annotated using AnnotSV [80] web server with neurofibromatosis-related HPO term identifiers: HP:0007524, HP:0005220, HP:0001067. This was followed by SV ranking based on SV Exomizer [47] score > 0.7 and ACMG_class score ≥ 3.

### 4.10. Estimation of Therapeutic Base Editing and Prime Editing Feasibility

Using the collected available nuclease information denoted in Table S14, we searched for DNA sequences upstream and downstream (±20 bases) of the target mutation that could be recognized as a PAM or TAM site within a functional distance of the target mutation. Lastly, we assessed potential bystander edits by checking whether any G>A substitutions within editing window would result in missense, splicing altering, or any other outcomes reported as pathogenic in the ClinVar database [21] (version 20240331). Prime editing was evaluated using web tool PE-designer [38] (http://www.rgenome.net/pe-designer/ (accessed on 19 January 2026)).

### 4.11. RNA Extraction and cDNA Synthesis

Total RNA was extracted from peripheral blood samples of the proband and both parents using the PAXgene Blood RNA Kit (Cat. No. 762174, PreAnalytiX GmbH, Zurich, Switzerland) according to the manufacturer’s protocol. RNA quality and concentration were assessed by spectrophotometry. First-strand cDNA was synthesized from total RNA using the ABScript Neo RT Master Mix for qPCR with gDNA Remover (Cat. No. RK20433, ABclonal Technology, Woburn, MA, USA). The cDNA product was diluted to 100 ng for subsequent RT-qPCR analysis.

### 4.12. RT-qPCR

Allele-specific expression analysis and relative quantification of *NF1* transcript levels were performed using the Power SYBR Green PCR Master Mix (Cat. No. 4367659, Applied Biosystems, Carlsbad, CA, USA) on an applied biosystem by life technology QuantStudio 6 Flex. Each 20 µL reaction contained 10 µL of 2× Power SYBR Green PCR Master Mix, 2 µL of cDNA template (100 ng), 1 µL each of forward and reverse primers (10 µM), and 6 µL of nuclease-free water. The thermal cycling conditions were as follows: initial denaturation at 95 °C for 10 min, followed by 40 cycles of 95 °C for 15 s and 60 °C for 60 s.

For allele-specific amplification, a *NF1* forward primer (5′-ACC CTC TCC TTG CCT CTT C-3′) was used in combination with either wild-type-specific reverse primer (5′-TAT TGG AAG CTG CTC GTC G-3′) or mutant-type-specific reverse primer (5′-GTG TTC TGC TGT CCT GCT C-3′). GAPDH was used as an endogenous reference gene with forward primer (5′-GGA AGC TTG TCA TCA ATG GAA ATC-3′) and reverse primer (5′-TGA TGA CCC TTT TGG CTC CC-3′). Each sample was analyzed in triplicate, and relative expression levels were calculated using the 2^-ΔΔCt method.

### 4.13. Statistical Analysis of mRNA Expression Levels in Pedigree

For allele-specific expression comparison (wild-type vs. mutant allele), statistical significance was determined using Student’s *t*-test. For comparative analysis of NF1 expression levels among family members (proband, paternal, and maternal samples), one-way ANOVA was performed followed by post hoc tests for multiple comparisons. Results are presented as mean ± standard error. *p*-values < 0.05 were considered statistically significant.

## Data Availability

The original data presented in the study are openly available in NCBI SRA at PRJNA1402113.

## Supplementary Materials

The following supporting information can be downloaded at https://www.mdpi.com/article/10.3390/ijms27031177/s1.
