# Supplementary material for: Neurofibromin 1 (NF1) Splicing Mutation c.61-2A>G: From Aberrant mRNA Processing to Therapeutic Implications In Silico
Source: Int J Mol Sci. 2026 Jan 23;27(3):1177. doi: 10.3390/ijms27031177 (PMC12898238; doi:10.3390/ijms27031177)
Supplement: Supplementary file 1 [file ijms-27-01177-s001.zip › FigS8_PAMTAM.pdf]

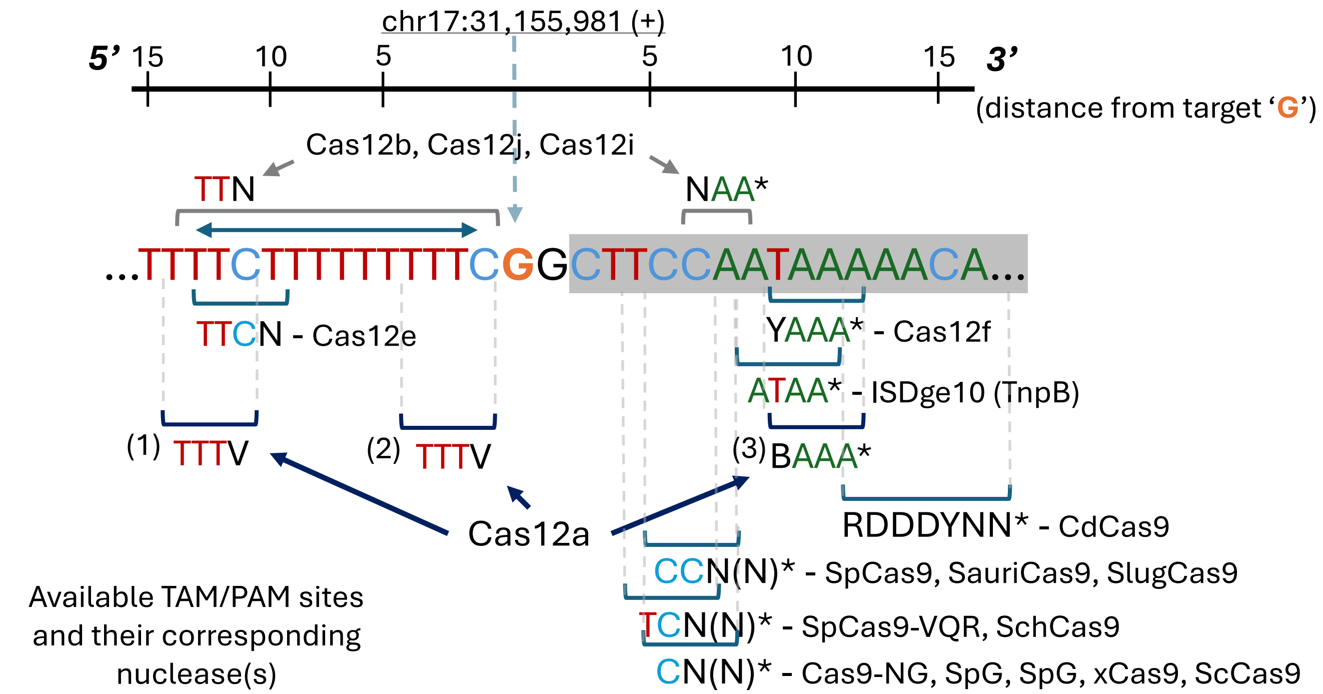

chr17:31,155,981 (+)
5' 15 10 5 5 10 15 3'
(distance from target 'G')
Cas12b, Cas12j, Cas12i
TTN
NAA*
...TTTTCTTTTTTTTTCGGCTTCCAATAAAAACA...
TTCN - Cas12e
YAAA* - Cas12f
ATAA* - ISDge10 (TnpB)
(1) TTTV
(2) TTTV
(3) BAAA*
Cas12a
RDDDYNN* - CdCas9
CCN(N)* - SpCas9, SauriCas9, SlugCas9
TCN(N)* - SpCas9-VQR, SchCas9
CN(N)* - Cas9-NG, SpG, SpG, xCas9, ScCas9
Available TAM/PAM sites
and their corresponding
nuclease(s)
