# Supplementary material for: Neurofibromin 1 (NF1) Splicing Mutation c.61-2A>G: From Aberrant mRNA Processing to Therapeutic Implications In Silico
Source: Int J Mol Sci. 2026 Jan 23;27(3):1177. doi: 10.3390/ijms27031177 (PMC12898238; doi:10.3390/ijms27031177)
Supplement: Supplementary file 1 [file ijms-27-01177-s001.zip › NF1_Supplementary_figure_captions.pdf]

Supplementary figure captions

Fig. S1. Patient's and unaffected family members' DNA short-read alignment centered at genomic location chr17:31, 155,981 (*NF1*, c.61-2A>G). Family members: KU10K-05706 – father, KU10K-05707 - mother, KU10K-05708 – healthy sibling of the NF1 patient, KU10K-05709 – NF1 patient.

Fig. S2. Small intronic *NF1* deletion in the patient and unaffected family members.

Fig. S3. Large intronic *NF1* deletion in the patient and unaffected family members. Family members: KU10K-05706 – father, KU10K-05707 - mother, KU10K-05708 – healthy sibling of the NF1 patient, KU10K-05709 – NF1 patient.

Fig. S4. Large intronic deletion in *KIT* gene – homozygous in the patient and heterozygous in unaffected family members. Family members: KU10K-05706 – father, KU10K-05707 - mother, KU10K-05708 – healthy sibling of the NF1 patient, KU10K-05709 – NF1 patient.

Fig. S5. RNA expression (log2-transformed TPM) across age groups, stratified by sex within the Korean general population. Error bars represent standard deviation. One-way ANOVA show a modest age effect ($p = 0.0068$), while no significant sex difference was observed ($p = 0.443$).

Fig. S6. Long-read DNA methylation shows novel CpG site at genomic location chr17:31,095,840 in the patient's sample

Fig. S7. NF1 mRNA expression quantified in the NF1 patient and patient's parents

Fig. S8. Available TAM/PAM sites and their corresponding nucleases
