# Supplementary figures and images for: Neurofibromin 1 (NF1) Splicing Mutation c.61-2A>G: From Aberrant mRNA Processing to Therapeutic Implications In Silico
Source: Int J Mol Sci. 2026 Jan 23;27(3):1177. doi: 10.3390/ijms27031177 (PMC12898238; doi:10.3390/ijms27031177)

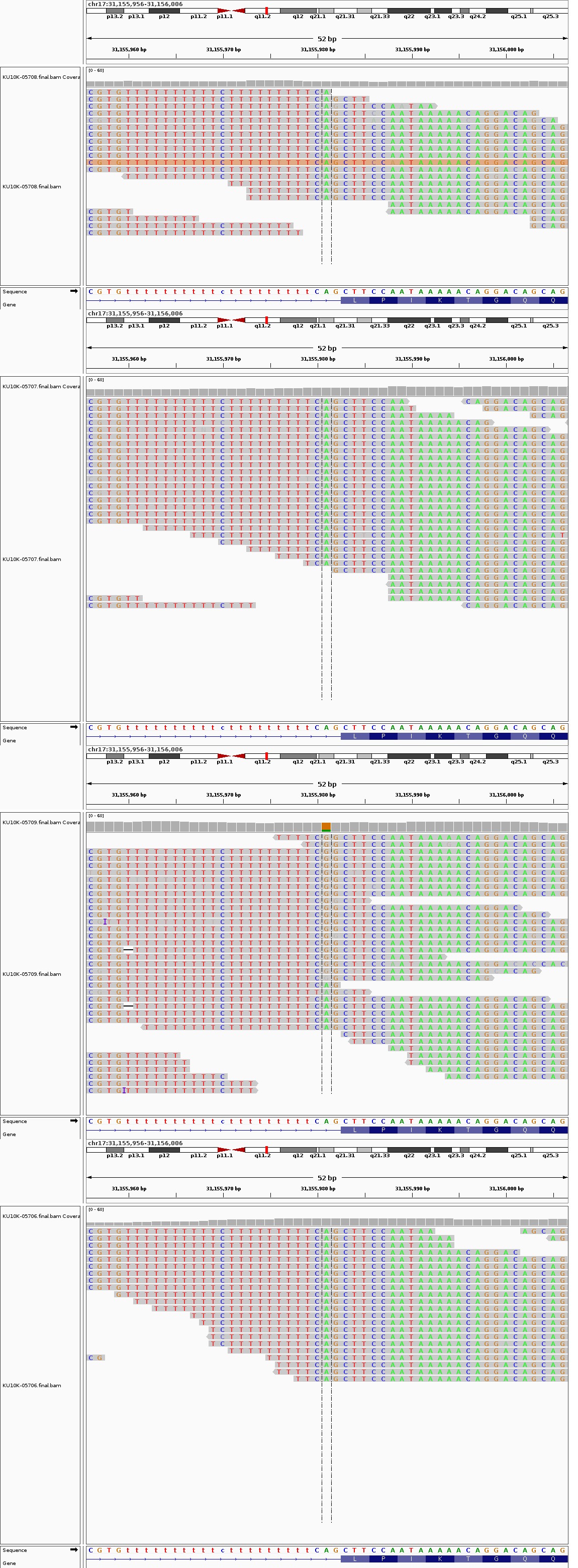

Supplement: Supplementary file 1 [file ijms-27-01177-s001.zip › FigS1_NF1-Family-31155981.jpg]

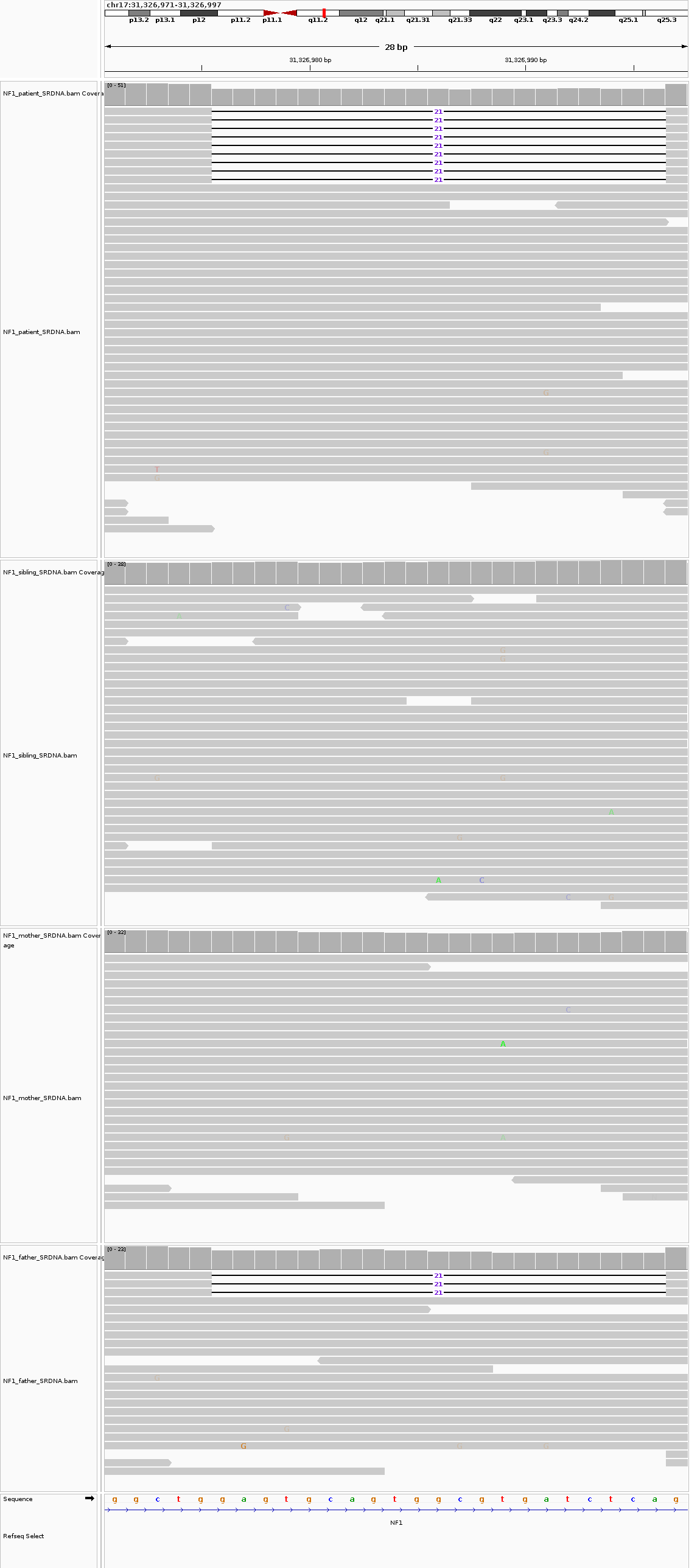

Supplement: Supplementary file 1 [file ijms-27-01177-s001.zip › FigS2_NF1_21DEL.png]

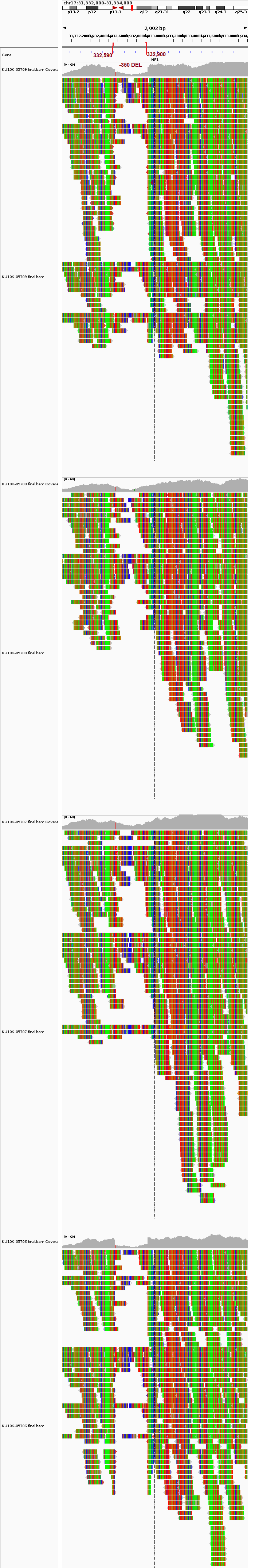

Supplement: Supplementary file 1 [file ijms-27-01177-s001.zip › FigS3_NF1_350DEL.png]

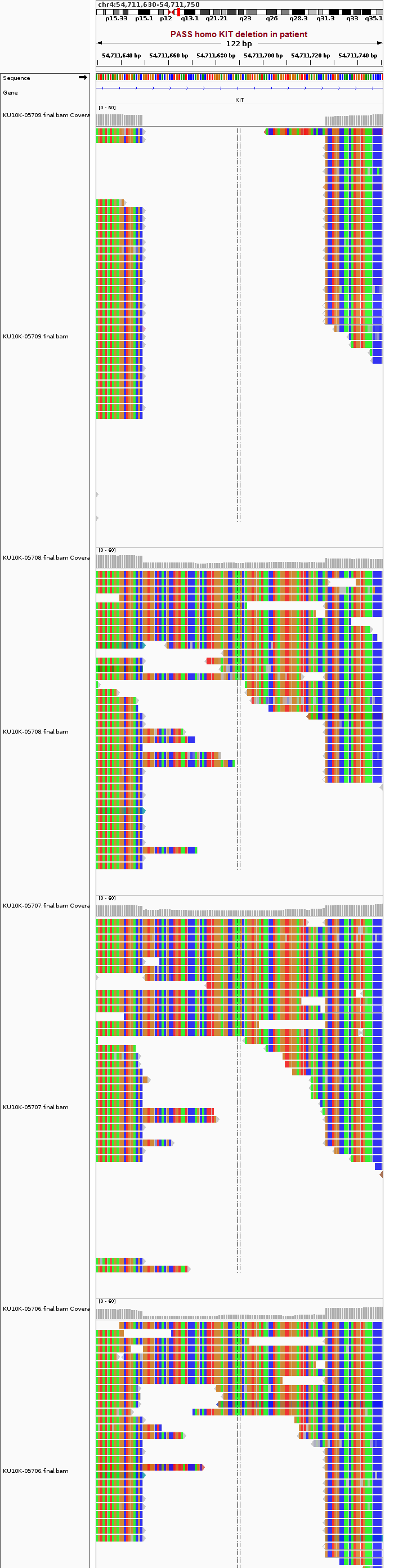

Supplement: Supplementary file 1 [file ijms-27-01177-s001.zip › FigS4_KIT_77DEL.png]

**NF1 Expression by Age Group and Sex (log2-transformed)**

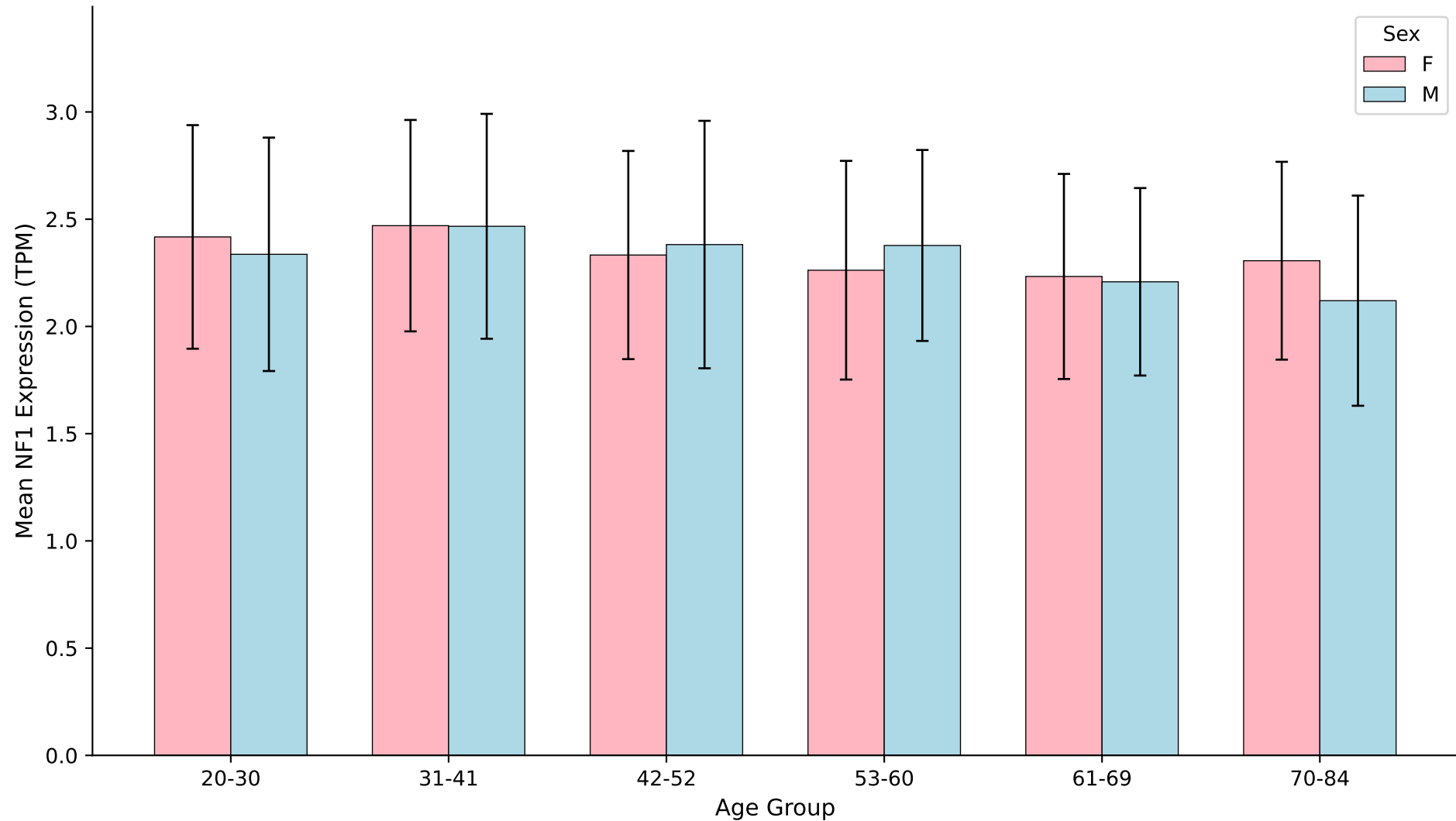

Supplement: Supplementary file 1 [file ijms-27-01177-s001.zip › FigS5_NF1Expression.pdf]

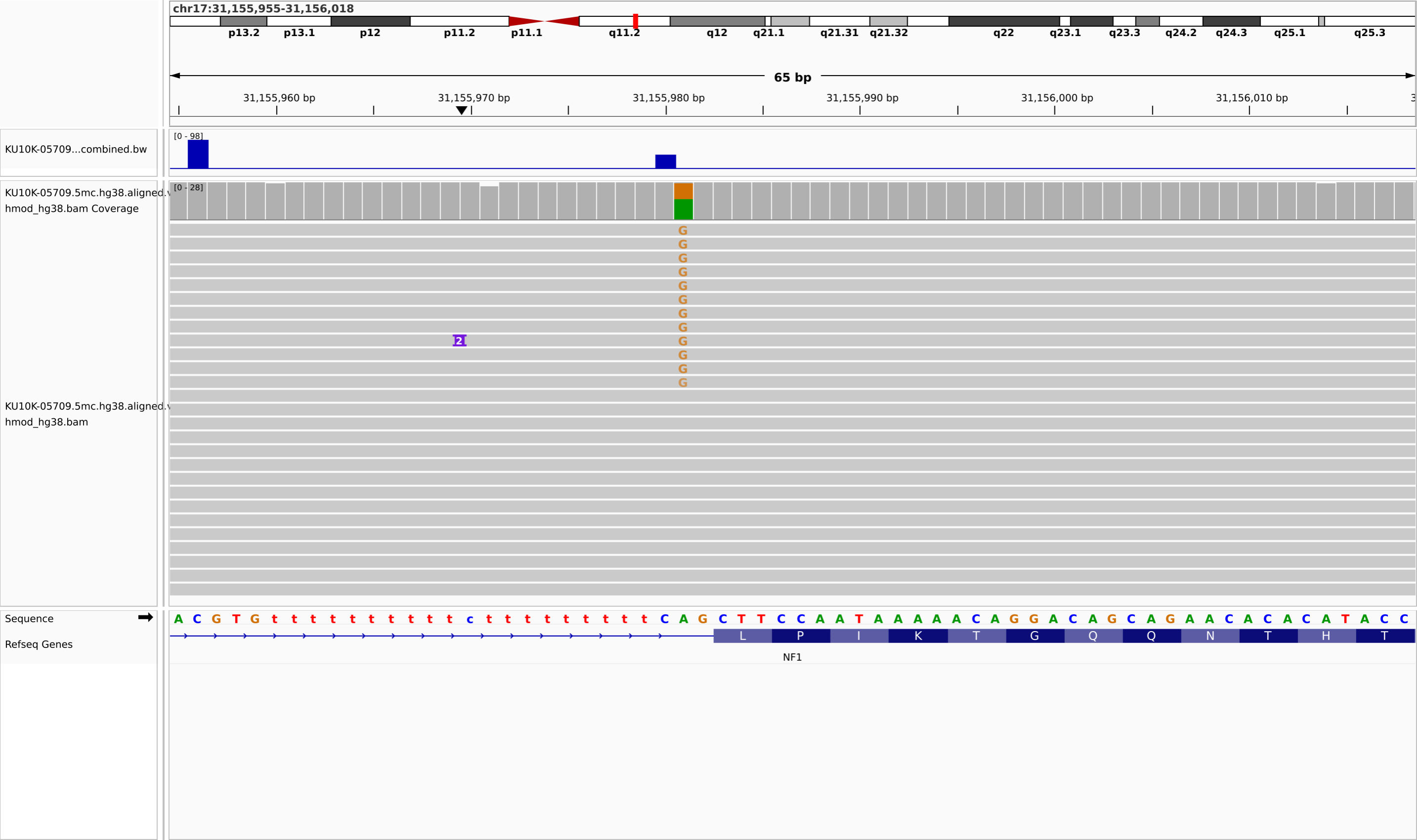

Supplement: Supplementary file 1 [file ijms-27-01177-s001.zip › FigS6_CpG.pdf]

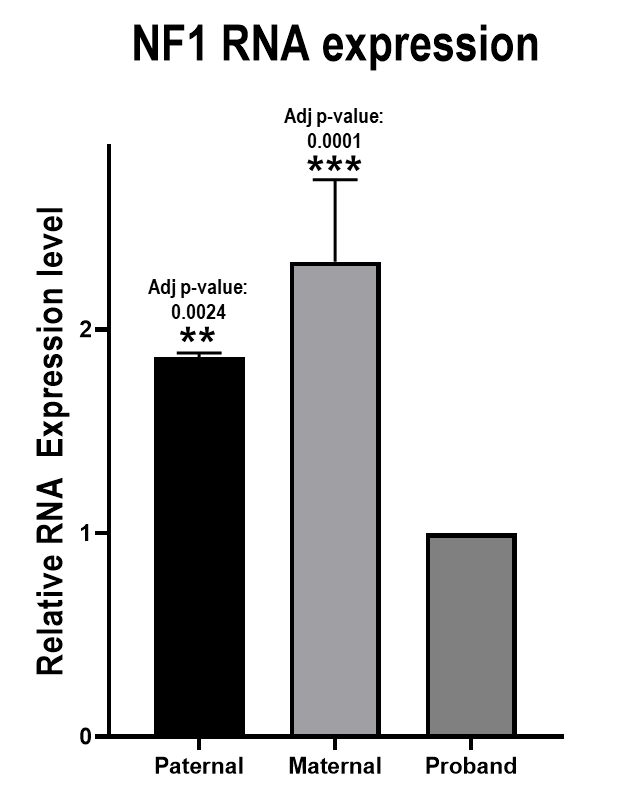

Supplement: Supplementary file 1 [file ijms-27-01177-s001.zip › FigS7_PCR.png]
